# RAN-related neural-congruency: a machine learning approach toward the study of the neural underpinnings of naming speed

**DOI:** 10.3389/fpsyg.2023.1076501

**Published:** 2023-06-20

**Authors:** Christoforos Christoforou, Maria Theodorou, Argyro Fella, Timothy C. Papadopoulos

**Affiliations:** ^1^Division of Computer Science, Mathematics and Science, St. John’s University, New York, NY, United States; ^2^Independent Researcher, New York, NY, United States; ^3^Department of Education, University of Nicosia, Nicosia, Cyprus; ^4^Department of Psychology and Center for Applied Neuroscience, University of Cyprus, Nicosia, Cyprus

**Keywords:** EEG, fixation-related potential (FRP), neural-congruency, machine learning, dyslexia, rapid automatized naming (RAN), RAN-related neural-congruency, eyetracking

## Abstract

**Objective:**

Naming speed, behaviorally measured via the serial Rapid automatized naming (RAN) test, is one of the most examined underlying cognitive factors of reading development and reading difficulties (RD). However, the unconstrained-reading format of serial RAN has made it challenging for traditional EEG analysis methods to extract neural components for studying the neural underpinnings of naming speed. The present study aims to explore a novel approach to isolate neural components during the serial RAN task that are (a) informative of group differences between children with dyslexia (DYS) and chronological age controls (CAC), (b) improve the power of analysis, and (c) are suitable for deciphering the neural underpinnings of naming speed.

**Methods:**

We propose a novel machine-learning-based algorithm that extracts spatiotemporal neural components during serial RAN, termed RAN-related neural-congruency components. We demonstrate our approach on EEG and eye-tracking recordings from 60 children (30 DYS and 30 CAC), under phonologically or visually similar, and dissimilar control tasks.

**Results:**

Results reveal significant differences in the RAN-related neural-congruency components between DYS and CAC groups in all four conditions.

**Conclusion:**

Rapid automatized naming-related neural-congruency components capture the neural activity of cognitive processes associated with naming speed and are informative of group differences between children with dyslexia and typically developing children.

**Significance:**

We propose the resulting RAN-related neural-components as a methodological framework to facilitate studying the neural underpinnings of naming speed and their association with reading performance and related difficulties.

## 1. Introduction

Rapid Automatized Naming (RAN), broadly defined as the ability to name as fast as possible visually presented stimuli such as colors, objects, digits, and letters (Kirby et al., 2010), is one of the most examined underlying cognitive factors of reading development and reading difficulties (RD). Indeed, since the original work by Denckla (1972), there has been an ongoing effort to explain the rather complex relationship between RAN and reading (e.g., Kirby et al., 2010; Araújo et al., 2015) across different ages (e.g., Landerl and Wimmer, 2008; Moll et al., 2009) and ability (e.g., Wolf and Bowers, 1999; Papadopoulos et al., 2009a; Torppa et al., 2013) groups and languages (e.g., Georgiou et al., 2012; Moll et al., 2014; Papadopoulos et al., 2021), focusing on group and individual differences. This effort has been based on behavioral/cognitive and neuroimaging data evidence. With regard to the former, two research approaches have been used, a componential (e.g., Georgiou et al., 2014) and a correlational (e.g., Papadopoulos et al., 2016) approach. With regard to the latter, data derived from fMRI studies (e.g., Cummine et al., 2015; Al Dahhan et al., 2020), electroencephalography (EEG) methods (e.g., Bakos et al., 2020) or more recently Fixation-Related Potentials (FRPs; e.g., Christoforou et al., 2021a). Although the evidence shows that RAN predicts reading performance and that RAN taps into universal cognitive mechanisms involved in reading (Papadopoulos et al., 2021), little is known about which neural components of RAN could better distinguish children with reading difficulties from typically developing peers. Thus, the present study aims to take this research line further: to produce and test methods that isolate the most informative neural components for RAN, suitable for deciphering group or individual differences in naming speed.

Based on correlational data, behavioral or cognitive research has repeatedly confirmed that RAN relates to reading because both tasks require serial processing and lexical access (e.g., Georgiou et al., 2013; Logan and Schatschneider, 2014). Also, it has been shown that RAN exerts direct effects on reading fluency only when oral reading fluency is the outcome measure (Georgiou et al., 2013; van den Boer et al., 2014; Papadopoulos et al., 2016), suggesting that articulation is essential for the RAN-reading relationship. Correlational research has also concluded that universal cognitive mechanisms such as working memory, attention, and processing speed are distal “common cause” processes to the RAN-reading relationship (Papadopoulos et al., 2016). Indeed, it is well-established that processing speed partly mediates the RAN-reading relationship (e.g., Bowey et al., 2005; Georgiou et al., 2012; Liao et al., 2015). Also, working memory is necessary because of the effortful nature of cognitive control required to perform naming speed tasks successfully, as it also occurs with word reading (Jacobson et al., 2011) or reading comprehension (e.g., Leong et al., 2008; Kendeou et al., 2012). Likewise, for serial processing to occur successfully, attention must be disengaged from naming a current item and directed to the next (Altani et al., 2017). Recent studies using eye-tracking methodology have verified the influential role of attention on RAN performance (e.g., Jones et al., 2009; Kuperman et al., 2016). Finally, evidence shows that speech production planning processes are also involved before articulation (e.g., Araújo et al., 2021).

These findings are further validated through research examining the unique contribution of articulation and pause time and what these components share with cognitive mechanisms such as the above. Since oral reading fluency and rapid naming require articulation alongside processing speed, the unique contribution of articulation time is justified (Georgiou et al., 2012). In turn, attention shifting, required as the participants move from one stimulus to another in a short time, is encapsulated in pause time (Wolf and Bowers, 1999; Georgiou et al., 2014), providing quick access to phonological codes or semantics in long-term memory (Rijthoven et al., 2018). Developmental data corroborate this evidence, as the contribution of pause time for typical readers decreases with time as they rely on larger orthographic units to read fluently (e.g., Georgiou et al., 2014). In contrast, pause time continues to explain significant variance in children with reading difficulties because of the deficits in accessing phonological codes experienced by this ability group (Ziegler et al., 2003; Araújo et al., 2011). Likewise, other processes have also been investigated, including multi-element sequence processing, coordinating rapid serial eye movements, and speech production planning processes of successive items (e.g., Gordon and Hoedemaker, 2016; Henry et al., 2018). However, studying these dimensions of the RAN-reading relationship was beyond the scope of the present paper to further explore their contribution.

Neurocognitive research has verified such findings with adults or typically developing and same-age poor readers, based on neuroimaging data. For example, Cummine et al. (2015), using functional magnetic resonance imaging (fMRI) with an adult group of typical readers, reported that RAN and reading rely on highly similar neural regions and that the RAN–reading relationship is driven by motor/serial processing. Likewise, Al Dahhan et al. (2020), using fMRI and eye-tracking methods, concluded that compared to typically achieving readers, readers with reading difficulties performed poorer in naming speed tasks. They had more extended articulation and pause times, longer fixation durations, and more regressions, resulting in decreased performance. This deficient processing was also reflected in greater bilateral activation and recruited additional regions involved with memory, namely the amygdala and hippocampus. Moreover, when the RAN-letter stimuli were visually or phonologically similar, adult readers showed higher activation in the amygdala and hippocampus, irrespective of their group (dyslexics vs. controls).

Furthermore, studies using eye-tracking (e.g., Easson et al., 2020) or electroencephalography (EEG) methods (e.g., Bakos et al., 2020) have provided additional evidence. Their results have focused on the RAN’s constituent components or the neurophysiological differences between children with reading difficulties and typically developing readers. For example, Easson et al. (2020) revealed significant contributions of fixation duration and saccade count to the prediction of naming speed performance. In addition, Bakos et al. (2020) showed that EEG activity differed between 10-year-olds with reading difficulties and their counterparts at around 300 ms after stimulus presentation. This difference was evident in the left-occipital-temporal P2 component and was statistically significantly correlated to RAN performance, albeit small *r*(72) = 0.24, *p* < 0.04.

More recently, Christoforou et al. (2021a,b) combined EEG and eye-tracking recordings to examine the underlying factors elicited during the serial Rapid-Automatized Naming (RAN) task that may differentiate between children with reading difficulties and chronological age controls (CAC). In doing so, the authors extracted fixation-related potentials (FRPs) under phonologically similar (rime-confound) or visually similar (resembling lowercase letters) and dissimilar (non-confounding and discrete uppercase letters, respectively) RAN tasks. As a result, the authors reported significant differences in FRP amplitudes between RD and CAC groups under phonologically similar and non-confounding conditions. These differences were evident in a cluster emerging around 128–170 ms in the frontal and occipital channels and between 80–160 ms for the rime-non-confusable and the rime-confusable RAN-letter tasks, respectively. However, no differences were observed in the case of the visual conditions. Moreover, regression analysis showed that the average amplitude of the extracted components significantly predicted RAN performance.

That research investigating the RAN-reading relationship concludes that RAN is a proxy for reading because it exerts similar processes to the neural reading system in the brain’s left hemisphere is not a surprise. This system includes a ventral stream that helps the reader recognize the words and their semantic meaning (Norton and Wolf, 2012) and a dorsal stream which connects sub-lexical phonological codes to orthographic representations (Pugh et al., 2001; Price, 2012). Deficits with the processing of grapheme-phoneme correspondence, in turn, are reflected in lower activation in the dorsal stream. Likewise, automatic visual word recognition deficits are reflected in lower activation in the ventral occipital-temporal system (Richlan et al., 2011). Consequently, when performing RAN tasks whose stimuli exhibit phonological or visual similarities, this network tends to suffer more (Al Dahhan et al., 2020; Christoforou et al., 2021a).

Despite these efforts to isolate the neural components for RAN, the findings about the different brain regions identified do not tell the complete story of the RAN-reading relationship. For example, the evidence does not tell us why group or individual differences exist or which are the most informative components that could help replicate such findings with groups of different ages, varying cognitive or linguistic abilities, or language. We argue that more advanced methods are needed to isolate the most informative components, explain group differences and improve the power of analysis. FRPs, for example, can be used as markers of ability, but we need more specific attributes to carry more information about group differences.

In recent years, machine learning approaches are becoming more prominent in analyzing EEG signals and studying neurocognitive processes. Machine learning allows an algorithm to isolate neural components that “optimally” characterize group differences under different conditions; therefore having the potential to detect more informative neural components than traditional EEG analysis methods (i.e., average ERPs and traditional frequency-band analysis). Several machine learning approaches have been proposed to overcome the methodological constraints of traditional EEG analysis methods. For example, single-trial correlation analysis (Christoforou et al., 2013) was developed to identify associations between continuous behavioral measures and concurrent neuronal activity. It was applied to exploring the neural underpinnings for the Stimulus Presentation Modality Effects in Traumatic-Brain-Injury treatment protocols. In the context of spatial cognition, a Common Spatial Pattern (CSP)-based single-trial analysis algorithm was proposed (Christoforou et al., 2018) to disambiguate the neural basis of two spatial-cognition processes, namely Perspective Taking and Mental Rotation. Machine-learning-based algorithms have been also proposed for decoding neural activity during complex interactions, such as consuming video and music context, toward studying user’s preferences and affective state (Dmochowski et al., 2012; Christoforou et al., 2017; Christoforou and Theodorou, 2021), as well as in other decision making (Philiastides and Sajda, 2005). In the context of reading and reading disorders, machine learning algorithms were proposed for detecting informative neural components during performing a phoneme elision task (Christoforou et al., 2022a,b), and classifying dyslexic from non-dyslexic participants during resting EEG (Rezvani et al., 2019). However, most of the proposed machine-learning approaches assume some prior domain knowledge of the spatial and temporal characteristics of the sought EEG components. They also require experimenter-controlled time-locked events (i.e., stimulus onset), and are typically limited to within-participant comparisons because of the large inter-subject variability in the EEG signals (Christoforou et al., 2010). These methodological requirements do not hold in the case of the serial RAN task which makes their direct application to RAN ineffective.

In the present study, we explore a novel machine-learning-based approach to isolate neural components informative of group differences between children with dyslexia and controls during the serial RAN. Our approach overcomes many methodological challenges of traditional methods which enable us to extract differential spatiotemporal profiles of neural components among children with dyslexia and controls during RAN and in the absence of experimenter-controlled time-locked events. Our method first formulates an optimization problem for extracting EEG components based on the Neural-congruency hypothesis. This relates to the premise that neural activity elicited during a cognitive task is similar (i.e., congruent) among participants that have mastered the task but less congruent otherwise (Christoforou et al., 2021b; Christoforou and Theodorou, 2021). Subsequently, our approach optimally combines the resulting components to identify neural differences between children with dyslexia and controls. We demonstrate the ability of our approach to extract informative neural components on a real EEG dataset involving children with dyslexia and controls of ages 9 and 12 (i.e., 3rd and 6th grade). Moreover, we examine the predictive power of the resulting components under a set of phonological and visual confounding RAN tasks. Importantly, our proposed analysis approach serves as a novel methodological framework for studying the neural underpinnings of cognitive processing in children under the serial RAN, on which traditional analysis methods have proven inadequate.

## 2. Materials and methods

### 2.1. Experimental task and data collection

The data we used in this study were collected as part of a broader project aiming to identify the neural underpinnings of dyslexia in children. In this section, we briefly describe the key parameters of the RAN experimental task and the data collection procedure relevant to our analysis; we refer to Christoforou et al. (2021a) for full details on the data collection apparatus.

#### 2.1.1. Participants

Participants were recruited from Grades 3 and 6 from inner-city public elementary schools in Cyprus. A total of 60 children (36 boys, 24 girls, age range = 7.6 through 12.1 years) participated in the study; all children were native Greek speakers. Two groups were formed from this sample: a group of children with dyslexia (DYS) and a chronological-age control group (CAC), based on a stepwise group selection process (see Christoforou et al., 2021a) using a lenient cutoff threshold on their reading fluency scores. Particularly, thirty Grade 3 and Grade 6 children (19 males; mean age = 9.6, SD = 1.5) who scored at least one standard deviation below their respective age group mean on the reading fluency tasks (word reading fluency and nonword reading fluency; ERS-AB; Papadopoulos et al., 2009c) and within the average range on verbal (Vocabulary Wechsler Intelligence Scale for Children—Third Edition; Greek standardization: Georgas et al., 1997) and non-verbal ability tasks (Nonverbal Matrices from the Cognitive Assessment System; Niglieri and DAS, 1997; Greek standardization: Papadopoulos et al., 2009b) were included in the DYS group. Another group of 30 children (17 males; mean age = 9.92 years, SD = 1.62) were randomly chosen from the same classes and were matched to the DYS group on chronological age and gender. Groups did not differ in age, *F*(1,58) = 0.22, ns, gender, χ2 (1, *N* = 60) = 0.28, ns, and the verbal and non-verbal ability measures, Wilks λ = 0.98, *F*(2,57) = 0.70, ns. Parental consent and school consent were obtained before to each assessment. The study was carried out per the Cyprus National Bioethics Committee recommendations (EEBK/EP/2011/10). It also received approval from the Ministry of Education and Culture, Cyprus (#7.15.01.27/17).

#### 2.1.2. Serial RAN task

**FIGURE 1:**
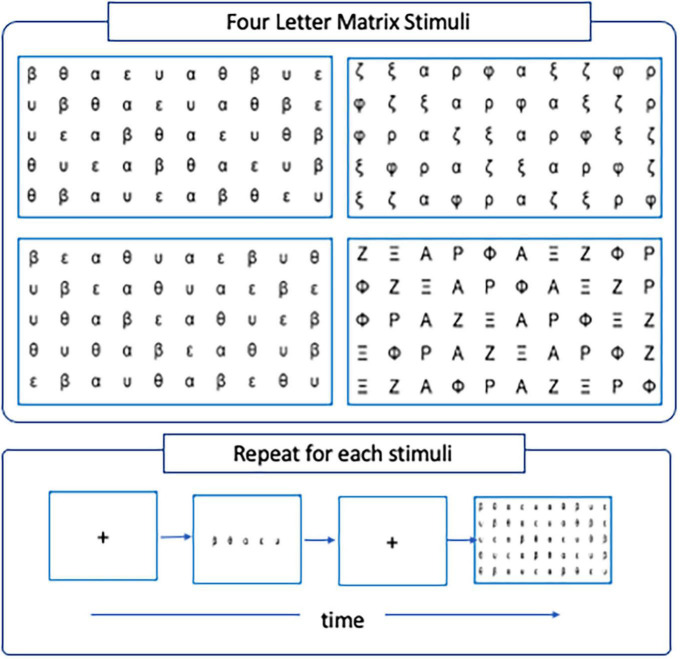
**(Top)** The four letter-matrix stimuli used in the serial RAN encapsulating the four experimental conditions, *Rime-confusable **(top-left)***, *Rime-non-confusable **(bottom-left)***, *Visual-confusable **(top-right)***, and *Visual-non-confusable **(bottom-right)***. ***(Bottom)*** Schematic representation of the serial-RAN trials, repeated for each one of the letter-matrix stimuli.

A computerized version of the serial Rapid Automatized Naming task was adapted from the work of Jones et al. (2008) to allow for simultaneous recordings of EEG and eye-tracking measurements during the experiment. The RAN task comprises four letter-matrix stimuli each encapsulating one of four conditions that differed by the degree of visual and phonological confusability among letters. In particular, the conditions encoded by the stimuli were *rime-confusable* (Condition 1), *rime non-confusable* (Condition 2), *visual confusable* (Condition 3) and *visual-non-confusable* (Condition 4). In the *rime-confusable* condition, pairs of letters that are phonologically confusable in the Greek alphabet (i.e., β-θ, ε-υ; beta-theta, epsilon-upsilon) were presented adjoining each other. In the *rime non-confusable* conditions, the pairs were disjoined (i.e., β–ε, β–υ, θ–ε, θ–υ, beta-epsilon, beta-upsilon, theta-epsilon, theta-upsilon). In the *visual-confusable* condition, pairs of letters that are visually confusable in the Greek alphabet (i.e., ζ-ξ, ρ-φ; zeta-xi, rho-phi), were presented adjoining each other. The visual similarity was removed in the *visual-non-confusable* condition by using the corresponding capital form of the letters (i.e., Z -Ξ, P -Φ). Each letter-matrix stimulus was organized in five rows and ten columns. Participants were shown the corresponding letter-matrix for each condition and asked to name each letter aloud, reading from left to right and from top to bottom, as fast and as accurately as possible. Before each conditioned stimulus, a fixation cross was displayed on the screen to prime participants to focus their eye-gaze at the center. The experimenter monitored the participants during the experiment and pressed the SPACE bar button the moment the participants name aloud the last letter of the letter-matrix. The experimenter also controlled the transition from one condition to the other. A schematic representation of the experimental task and example stimuli is shown in Figure 1.

#### 2.1.3. EEG and eye-tracking data collection during RAN

All participants had to perform the serial RAN task while simultaneous eye-tracking and EEG measurements were collected during the session. Eye-tracking data were collected using the EyeLink 1000 Plus eye-tracker (SR Research, Kanata, ON, Canada) at a 1,000 Hz sampling rate. Eye fixations and saccade events were automatically detected and recorded by the EyeLink parser along with the raw gaze data. The stimuli were presented on a Dell Precision T5500 workstation with an ASUS VG-236 monitor (1,920 × 1,080, 120 Hz, 52 × 29 cm) at a viewing distance of 60 cm. A chin rest was used to maintain the participant’s head proper positioning and to improve measurement stability. A nine-point calibration session was performed prior to experiment to establish a correct mapping to screen coordinates. EEG data were collected using a BioSemi Active-two system (BioSemi, Amsterdam, Netherlands) at a sampling rate of 256 Hz. Before to the experimental session, a 64-electrode cap was fitted to the participants, following the 10/20 system. The DC offset of all sensors was kept below 20 mV using electro gel. To align the stimulus presentation time to the EEG and eye-tracking signal streams, event markers were sent to each device at the beginning and ending of each condition. Specifically, the event markers were sent to the trigger channel of the EEG amplifier via parallel port TTL signals and to the eye-tracking recorder via direct ethernet event logs. Eye-tracking data were collected for each participant in a separate data files; specifically an EDF file format (default of the eye-link trackers), and the EEG data in a BDF file format.

### 2.2. EEG and eye-tracking data pre-processing

#### 2.2.1. EEG data pre-processing

All EEG data preprocessing was implemented using custom python code and the MNE python library. Preprocessing of the EEG signals was performed separately on the recordings of each participant. First the raw, continuous EEG data and the corresponding trigger channel were loaded from the data file (i.e., BDF data format file) using MNE library functions. Once loaded, all EEG data channels were re-referenced to the average channel. A 0.5 Hz high pass filter was used to remove DC drifts and a notch filter at 50 Hz and 100 HZ was used to reduce the power-line noise interferences. Markers in the EEG trigger channel were used to identify the timestamp of the beginning and end of each stimulus trial (i.e., each condition). Four EEG sub-segments were generated for each condition, each segment spanning 2 s before each stimulus onset to 2 s after the trial conclusion (i.e., after the participant finished reading all letters in the letter-matrix). The baseline amplitude of each segment (i.e., activity from −200 ms to zero) was subtracted from each segment. After basic EEG pre-processing, we have an EEG segment *EEG*_*p,c*_ for each participant p and condition *c*, each representing the entire EEG recording of that participant reading the entire letter-matrix of that conditions. It is important to note that the duration of each EEG segment varies from condition-to-condition and from participant to participant, as each participant took a different time to complete the reading of each matrix.

#### 2.2.2. Eye-tracking data preprocessing

Preprocessing of the eye-tracking data was also performed separately for each participant. The eye-fixation data and the corresponding event logs were loaded using the PyGaze Analyzer python library. Information on the event logs was used to determine the timestamp of the beginning and ending of each stimulus trial. Each eye-fixation data point comprised an absolute timestamp, the x-y screen coordinates of the fixation and durations in milliseconds. The set of eye-fixation points was grouped into four subsets, one for each of the four conditions. Each fixations subset comprised those fixations whose timestamp fell within the time window spanning the beginning and end of the stimulus presentation of that condition. The timestamp of the stimulus onset event of each condition (as recorded in the eye-tracking data) was subtracted from the timestamp of each fixation within that condition’s fixation subset to achieve temporal alignment between the fixation data to the EEG data. Therefore, each fixation’s timestamp is now relative to the onset of the stimulus.

### 2.3. Generating single-trial fixation-related potentials

A particular challenge in analyzing EEG signals obtained during the serial RAN is the lack of experimenter-controlled, time-locked trials necessary to extract Event-related Potentials. As such, we opted to explore the neural activity time-locked to the onset of eye fixations; this activity is referred to as single-trial Fixation-related Potential (sFRP). To extract the sFRP, we integrate information from eye-tracking and EEG measurements. In particular, the onset of each eye-fixation’s timestamp in the dataset is used as a temporal marker to epoch the EEG segments. More precisely, given an EEG data segment *EEG*_*p,c*_ (i.e., the segment generated during the EEG pre-processing step above, which represents the EEG response of participant *p* while reading the entire letter-matrix of condition *c*), and given the corresponding set of fixations *FIX*_*p,c*_, we epoch the *EEG*_*p*,*c*_ between −200 ms to 500 ms of the onset time of each *f* ∈ *FIX*_*p*,*c*_ and subtract the baseline amplitude of each epoch. This procedure results in a new set defined as


$$F\,R\,P_{p,c}={\left\{s\,F\,R\,P_{i}\right\}}_{i=1}^{\left|F\,I\,X_{p,c}\right|}$$

where *sFRP_i_* corresponds to the EEG epoch on the onset time of the i-th fixation of *FIX*_*p,c*_. We note that the two sets have the same cardinality. The generation of all sFRPs was implemented using custom python code.

### 2.4. Reading-related neural-congruency components

Our objective was to isolate neural components in the extracted fixation-related potentials that were likely modulated by RAN tasks and were informative of differences between CAC and DYS. Our approach was motivated by the hypothesis that the neural activity of participants that have developed adequate reading skills would exhibit neural activation patterns congruent with other participants with adequately developed reading skills. While contrarily, participants who experienced reading difficulties would have neural responses that deviated from such stereotypical patterns. Toward this objective, we formulated an optimization procedure to isolate neural components congruent among participants with sufficiently developed reading skills and explore those components as potential differentiation neuro-markers between CAC and DYS. Here, we provide details of our approach to isolate such reading-related neural-congruency components.

We seek to identify components (i.e., spatial projections of the fixation-related potential) that capture neural activity that maximally correlates among a group of children with adequately developed reading skills (i.e., CAC group). For this, we formulate an optimization problem as follows: for a group of S participants, 𝒮 = {*s*_1_, *s*_2_, …, *s*_*S*_}, *where s*_*i*_ ∈ ℤ^+^ denotes a participants index, representing a CAC group, we define the *between-subject* and *within-subject* cross-covariance matrices was :


$$R_{b}=\frac{1}{S\,\left(S-1\right)}\,\sum_{i\in 𝒮}\sum_{j\in 𝒮}\left(1-\delta_{i\,j}\right)\,{\text{R}}_{i\,j}$$

$${\text{R}}_{w}=\frac{1}{S}\,\sum_{i\in 𝒮}R_{i\,i}$$

where


$${\text{R}}_{i\,j}=\frac{1}{K}\,{\text{X}}_{i}\,{\text{X}}_{j}^{T}$$

where *K* is a normalizing scalar, δ_*ij*_ is the Kronecker delta^1^, and *X*_*s*_ ∈ ℝ^*D* × *S*.*F*^ is the horizontally concatenated matrix comprised of the fixation-related potential of a participant *s* during a given condition (i.e., reading of a letter-matrix stimulus), defined as:


$${\text{X}}_{s}=\left[s\,F\,R\,P_{1},s\,F\,R\,P_{2},s\,F\,R\,P_{3},\ldots ,s\,F\,R\,P_{F}\right]$$

For a spatial projection vector **w** ∈ ℝ^*D*^, the average Pearson Product Moment Correlation Coefficient between the fixation-related potentials, projected onto vector **w**, across every pair of participants in the group is then defined as:


$$\rho =\frac{{\text{w}}^{T}\,{\text{R}}_{b}\,\text{w}}{\left({\text{w}}^{T}\,{\text{R}}_{w}\,\text{w}\right)}$$

We consider ρ as a measure of the degree of congruency in reading-related neural activity (projected onto component **w**) among participants with adequately developed reading skills. As such, we seek to find the component **w** that maximized ρ. Taking the derivative of ρ with respect to **w** and setting it to zero, we get the solution of the optimization given as the eigenvectors to the generalized eigenvalue problem:


(1) $$\left(R_{w}^{-1}\,R_{b}\right)\,{\text{w}}_{k}=\lambda_{k}\,{\text{w}}_{k}$$

where **w_k_** is the k-th eigenvector of the matrix $\left(R_{w}^{-1}\,R_{b}\right)$ and corresponds to the component (i.e., spatial projection vector) that captures the k-th largest correlation in neural activity, and λ_*k*_ is the corresponding eigenvalue and denotes the strength of the correlation. We note that since $\left(R_{w}^{-1}\,R_{b}\right)$ is a *D* × *D* matrix (D being the number of channels), there are *D s*olutions to the optimization problem (i.e. the D eigenvectors), each identifying a component at different correlation strength, with the first eigenvector (i.e., *k* = 1) having the strongest correlation, and subsequent components appearing in descending order of correlation strength. As such, the vector **w**_1_defines a component (spatial projection) where neural activity is most strongly correlated among participants in the adequately developed reading skills, **w**_2_ defines the component where neural-activity exhibits the second strongest correlation among the groups, and so on.

To determine the reading-related neural-congruency (RRNC) score of an individual participant *s* with respect to the kth component, we measure the correlation between the fixation-related potentials of the subject to the fixation-related potentials of each subject in the group, after projecting both onto the component **w**_*k*_. Formally, we define the reading-related neural congruency score for a participant s and a component **w**_*k*_ as:


$$R\,R\,N\,C_{s,k}=\frac{{\text{w}}_{k}^{T}\,{\text{R}}_{s}^{b}\,{\text{w}}_{k}}{{\text{w}}_{k}^{T}\,{\text{R}}_{s}^{b}\,{\text{w}}_{k}}$$

where


$${\text{R}}_{s}^{b}=\frac{1}{S}\,\sum_{i\in 𝒮}R_{s\,i}+R_{i\,s},{\text{R}}_{s}^{w}=\frac{1}{S}\,\sum_{i\in 𝒮}R_{s\,s}+R_{i\,i},$$

We calculate RRNC scores separately for each condition (i.e., Rime-confound, Rime-non-confound, Visual-confound, Visual-non-confound). Moreover, to avoid training bias during the component extractions, data from the subject to be tested were excluded from the component extractions step. Finally, we define the neural-congruency feature vector of each participant s as:


(2) $${\text{c}}_{s}={\left[R\,R\,N\,C_{s,1},R\,R\,N\,C_{s,2},\ldots \,R\,R\,N\,C_{s,K}\right]}^{T}$$

Each *RRNC*_*s*,*k*_ score measures the strength of the congruency in neural activity between participant s and the CAC group, with respect to the *k*-th component. Therefore, the neural-congruency feature vector **c**_*s*_ encapsulates the neural-congruency of participant *s*, across all *K* components. In essence, the feature vector **c**_*s*_ characterizes the overall congruency observed in the participant’s neural activity for each extracted component. We note that the dimensions of the vector **c**_*s*_ are indexed in descending order, according to the lambda score of each extracted component.

### 2.5. Aggregation of RAN-related neural-congruency components

Our goal was to explore whether information captured in the feature vector of neural-congruency components **c_s_** is predictive of differentiating between DYS and CAC groups during the RAN task. Toward this objective, we considered two approaches for aggregating the RRNC scores into determining markers of dyslexia. The two approaches are detailed below.

#### 2.5.1. Cumulative RAN-related neural-congruency metric

The first approach defines a neural metric by simply summing the RRNC score corresponding to the first $\hat{K}$ components in the neural-congruency feature vector (i.e., those with the highest variance). Formally, given a feature vector **c**_*s*_ of a participant *s* (as defined in eq. 2), we define the Cumulative RAN-related Neural-congruency metric (C-RRNN) as follows:


$$C-R\,R\,N\,N_{s}=\sum_{k=1}^{\hat{K}}{\text{c}}_{s}\,\left(k\right)$$

where the index *k* denotes the k-th element of the vector. The value of $\hat{K}$ = 3 was selected by identifying the ‘knee’ in the plot of eigenvalues of equation 1, and was fixed across the calculation of C-RRNN of all participants.

#### 2.5.2. LASSO-weighted RAN-related neural-congruency metric

The second approach learns a classifier that optimally weights the contribution of each of the identified neural congruency components to best differentiate between typically developing children and children with dyslexia. In particular, we employed a sparse Logistic Regression classifier with LASSO regularization, using the *K* = 10 components with the highest lambda values as independent variables, and an individual’s group (DYS or CAC) as the dependent variable. We opted to use a sparse classifier because it minimizes the number of non-zero parameters, thus, favoring selecting a small subset of meaningful neural-congruency components. The classifier’s prediction output corresponds to an optimally weighted-sum of the individual neural-congruency scores that maximizes the differentiation between DYS and CAC. Moreover, since the optimal weights are calculated using the LASSO regularizations, we refer to the resulting prediction score as LASSO-weighed RAN-related Neural-congruency metric. In our analysis, a separate classifier was trained on each of the four conditions (i.e., Rime-confusable; Rime Non-confusable; Visual-confusable; Visual Non-confusable) using a leave-one-participant-out cross-validation procedure to avoid training biases.

### 2.6. Spatiotemporal profiles of RAN-related neural-congruency

Given the solutions to the generalized eigenvalue problem, the temporal profile of each component was calculated as the product of each component ${\hat{\text{w}}}_{k}$, with each single-trial response and taking the grant-average response of the projected components. Moreover, the topographical profile (i.e., the forward model) of each component was calculated as follows:


$${\text{a}}_{k}=\frac{{\text{R}}_{w}\,{\hat{\text{w}}}_{k}}{{\hat{\text{w}}}_{k}^{T}\,{\text{R}}_{w}\,{\hat{\text{w}}}_{k}}$$

The forward model captures the covariance between each component’s activity as measured by each electrode.

### 2.7. Statistical analysis

To avoid training bias, all model parameters, including the extracted neural-congruency components and classifier weights, are trained using a leave-one-participant-out cross-validation procedure. The classifier’s generalization performance is calculated as the area under the Receiver Operator Characteristic curve (AUC) on cross-validated scores. A permutation test is used to determine statistical significance levels over AUC scores (10,000 repetitions). A two-way ANOVA was used for group and grade comparisons, with the neural congruency metrics as the dependent variable.

## 3. Results

### 3.1. Group comparisons using the RAN-related neural-congruency metrics

**FIGURE 2:**
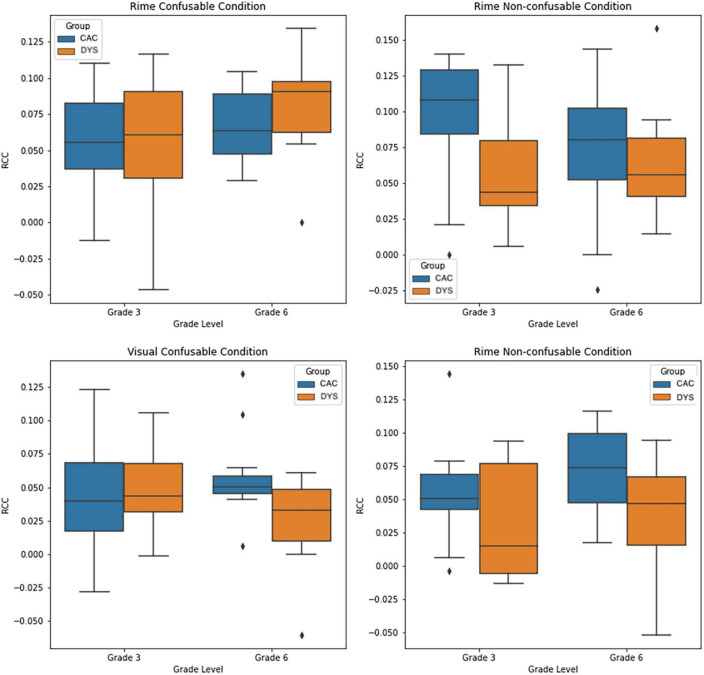
Box-plot showing the distribution of the Cumulative RAN-related Neural Congruency scores for each experiment condition, and each group.

For each participant, Cumulative Neural-Congruency scores were calculated as the sum of the three components with the highest lambda values, corresponding to the components whose projection has the highest correlation. To avoid training biases during the neural-congruency component identification, data from the participant for whom the Neural-Congruency score was to be calculated was excluded from the component identification step. Neural-Congruency scores were obtained and analyzed separately for each of the four conditions (i.e., Rime-confusable; Rime Non-confusable; Visual-confusable; Visual Non-confusable). For each condition, a separate two-way ANOVA was performed to analyze the effect of group (i.e., CAC vs. DYS) and grade (grade 3 vs grade 6) on the Neural-Congruency scores. A two-way ANOVA on Rime-confusable Cumulative RAN-related Neural-Congruency scores shows a significant main effect of grade (*p* < 0.04), with grade 6 group showing higher neural-congruency than the grade 3 group. The analysis also releveled there was not a significant interaction effect between the group and grade, *F*(1,52) = 0.740, *p* = 0.39, nor a statistically significant effect for group (*p* = 0.19). A two-way ANOVA on Rime non-confusable Neural-Congruency scores revealed a significant main effect of group (*p* < 0.01). Participants in the control group showed a higher Cumulative RAN-Related Neural-Congruency scores than the participants with dyslexia group. There was no main effect of grade (*p* = 0.61), and there was no statistically significant effect observed between group and grade *F*(1,52) = 1.67, *p* = 0.20. On the Visual Confusable task, a two-way ANOVA revealed a statistically significant interaction effect between the group and grade *F*(1,52) = 4.22, *p* = 0.04. The analysis also revealed there was not a statistically significant main effect of either the group or the grade (*p* > 0.05). Finally, the Visual non-confusable task revealed a main effect of the group (*p* < 0.01), with participants in the control group showing a higher Neural-congruency scores than the children with dyslexia group (DYS). No grade or interaction effect between grade and groups were observed during the Visual non-confusable. Figure 2, shows the box plots for the four two-way ANOVA for each condition. Moreover, a two-factor repeated measures ANOVA was performed to compare the effect of modality condition (rime vs visual) and confusability (confusable vs non-confusable). The analysis showed a statistically significant difference in RAN-related Neural-Congruency scores between modality conditions (*p* < 0.001) with the Rime modality exhibiting higher scores.

### 3.2. Lasso-weighted RAN-related neural-congruency as a significant predictor of group differences across conditions

**FIGURE 3:**
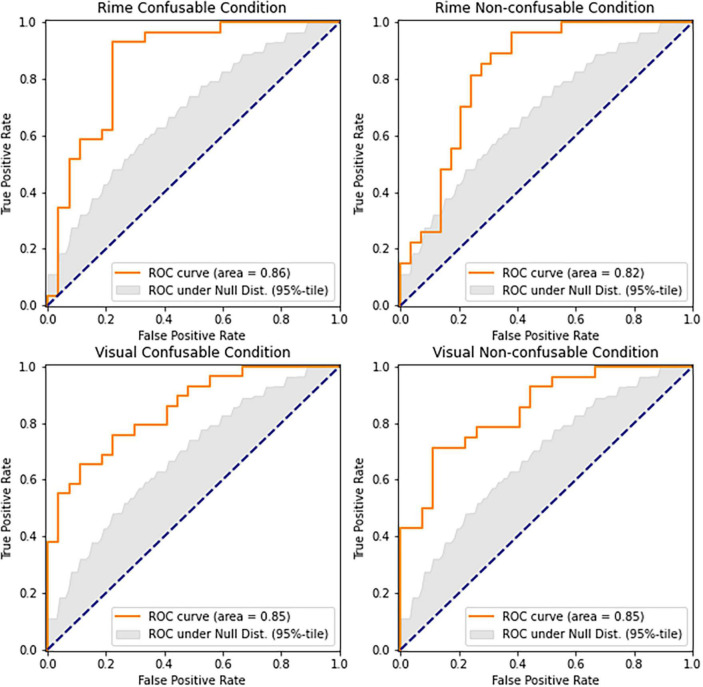
Shows the ROC curves of the predictions based on the LASSO-weighted RAN-related Neural-congruency components. The gray area denotes the ROC score under the null hypothesis (i.e., neural-congruency scores between DYS and CAC groups are indistinguishable). All four graphs show that the LASSO-weighted RAN-related Neural-congruency scores carry significant predictive information as the condition (i.e., DYS or CAC) of the participants.

**FIGURE 4:**
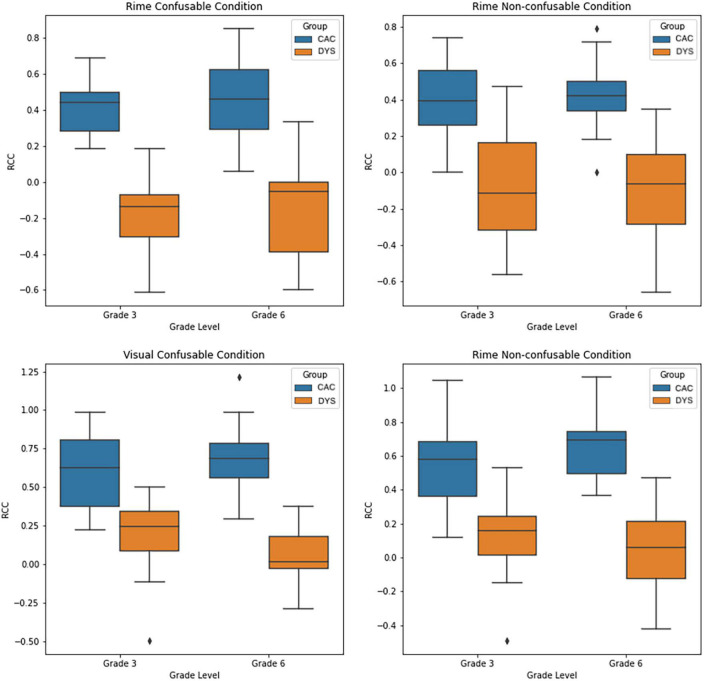
Box-plot showing the distribution of the LASSO-weighted RAN-related Neural Congruency scores for each experiment condition, and each group.

We aimed to further explore the characteristics of the underlying neural activity captured by the neural-congruency components and determine the degree to which each neural-congruency component contributes toward inferring an individual’s group (i.e., DYS or CAC). We hypothesized that a weighted aggregation of individual neural-congruency components would carry predictive information about the participant’s condition. We employed a sparse logistic regression classifier using the ten components with the highest lambda values as independent variables, and an individual’s group (DYS or CAC) as the dependent variable. The classifier was modeled and trained according to the procedure described in Section “2.5.2. LASSO-weighted RAN-related neural-congruency metric.” The statistical significance levels over AUC scores were established using a permutation test (10,000 repetitions). In all four conditions, the cross-validated AUC scores of the classifiers show that the LASSO-weighted RAN-related Neural-congruency metric predicted an individual’s group. The prediction accuracy for all conditions, the Rime-confusable condition (AUC = 0.86, *p* < 0.00001), Rime Non-confusable condition (AUC = 0.86, *p* < 0.00001) the Visual Non-confusable (AUC = 0.81, *p* < 0.00001), and the Visual Confusable condition (AUC = 0.73, *p* < 0.005) was high and statistically significant. The ROC curves and corresponding AUC scores for each condition and the 95th-percentile envelop of the ROC curve under the null-distribution are depicted in Figure 3. The boxplot in Figure 4 shows the distribution of the lasso-weighted RAN-related neural-congruency for each grade and group and experimental condition.

### 3.3. Spatio-temporal profile of neural-congruency components

**FIGURE 5:**
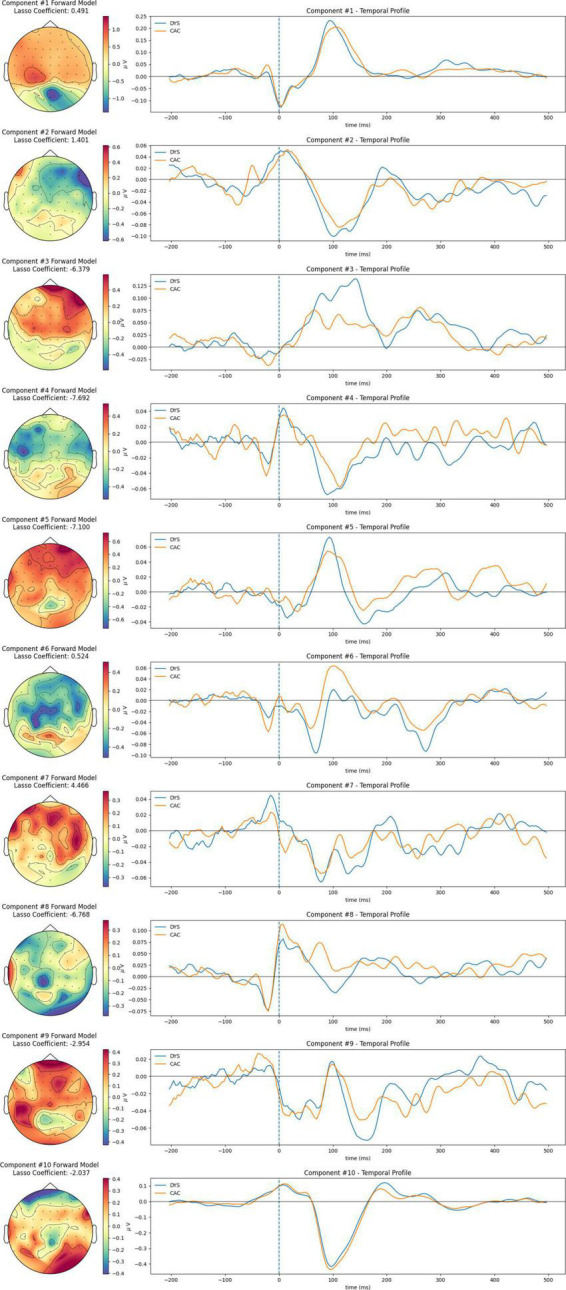
Spatiotemporal profiles of RAN-related Neural-congruency component for the rime-confusable condition; The corresponding plots for all conditions are provided in the Supplementary material.

The spatio-temporal profile of each RAN-related Neural Congruency for all conditions is shown in Figure 5 and the Supplementary material. Each spatio-temporal profile comprises the “Forward model,” which shows the spatial distribution of the correlated neural activity captured by the corresponding component, and the temporal profile–the time course of the FRP’s neural activity when projected onto that neural-congruency component. Visual inspection of the temporal profile provides insights into timeframe differences between groups and condition intensify. Similarly, visual inspection of the forward model alludes to potential brain areas from which the underlying neural activity originated from. The weights associated with each channel in the forward model capture the electrical coupling of the correlated components. The components are ordered based on their corresponding lambda scores, with component #1 reflecting the highest lambda, and component #10 the smallest lambda value. The LASSO-coefficients associated with each component are shown over each component’s forward model and indicate the weight used to aggregate the neural-congruency components.

### 3.4. Naming speed behavioral data analysis

Analysis of the behavioral data obtained during the experiment has been previously reported by Christoforou et al. (2021a) and it is outside the scope of this papers. However, to provide the context on our results on neurophysiological data, we include a summary of the behavioral data analysis of this experiment. A MANOVA analysis was performed on the behavioral data, with the naming speed performance time for each of the four RAN tasks as dependent measures and Group (2) as a fixed factor. The main group effects was significant, Wilks’ Lambda = 0.754, *F*(4,55) = 4.48, *p* < 0.01, η^2^ = 0.20. Subsequent univariate analyses demonstrated that the group’s main effect was significant for all individual measures after Bonferroni adjustments (Supplementary Table 1). The DYS group performed significantly poorer than the chronological age controls in all naming speed measures.

## 4. Discussion

Methodological difficulties in using traditional neurophysiological techniques to investigate the neural underpinning of dyslexia during serial RAN have hindered the development of studies in that direction (Bakos et al., 2020; Christoforou et al., 2021a). To help alleviate this problem, we proposed a novel computational approach for identifying neural components elicited during the serial RAN task. We also explore the component’s contribution to characterizing the underlying neural differences between children with dyslexia and chronologically age controls in four experimental conditions. Specifically, we formulated an optimization problem to extract spatiotemporal components from EEG measures that maximize the correlation between single-trial fixation-related potentials during serial RAN. We treated RAN as an additional variable to the diagnosis of reading difficulties, explaining the shared variance of the disorder. Based on the resulting components, we defined the per-subject neural-congruency scores that indicate the degree to which each participant engaged in neural processes relevant to the RAN task. Results show that the neural-congruency components capture the neural activity of cognitive processes associated with reading and are informative of group differences between children with dyslexia and typically developing children. Moreover, our results provide insights into the spatial and temporal characteristics of the underlying mental process involved in the naming speed and points to which potential neuro-cognitive mechanisms differentiate between children with and without dyslexia. These findings are robust given the careful matching of the participating groups based on their verbal and non-verbal ability and demographic variables. Furthermore, the study findings contribute to the relevant research because previous evidence has overlooked the contribution of neurophysiological measurements during serial RAN tasks and their relation to behavioral measures (i.e., naming speed) that together might explain reading performance and related difficulties.

### 4.1. Differences between DYS and CAC in the cumulative RAN-related neural-congruency components

Regarding the contribution of the Cumulative RAN-related neural-congruency components in differentiating between children with and without dyslexia, the results revealed significant differences between the groups in the both non-confusable conditions (i.e., Rime non-confusable, and Visual non-confusable). Specifically, on the one hand, cumulative RAN-related neural-congruency scores in typically developing children were significantly higher than their counterparts in the DYS groups. This finding denotes an increase in the synchronicity in neural activity in the CAC groups, which suggests that the CAC group has developed a more consistent stereotypical response in neural activations when engaging processes associated with the execution of the serial RAN task. Breznitz and colleagues (Breznitz, 2001, 2003; Breznitz and Misra, 2003) have proposed the ‘synchronization hypothesis’ to describe this phenomenon. According to this hypothesis, accurate information integration in decoding words can occur only when the modalities and brain systems are synchronized. This synchronization, therefore, requires that the processing speed and the accuracy with which content information is processed and transferred within and between the various activated neural systems are readily available. Our findings further confirm this hypothesis that this synchronicity in neural activity is evident in typically developing readers but to a lesser degree in children with dyslexia.

Indeed, on the other hand, the consistency in the synchronicity of the responses diminishes across the DYS groups, suggesting a lack of regularity in the processing the letter stimuli in the serial RAN. Visual inspection of the time course of the three neural-congruency components used in calculating the Cumulative RAN-related neural-congruency score suggests that the difference in congruency appears between 100 ms–200 ms following the fixation onset. The analysis also shows that the effect that emerged in the non-confusable tasks is not present in the confusable tasks (i.e., Rime-confusable, and Visual-confusable). That is, there were no significant differences observed in the Cumulative scores between DYS and CAC. We interpret these effects in the context of neural efficiency theory. Specifically, we argue that the CAC group has developed efficient mechanisms for recognizing, decoding, and reading letters as captured by the consistency in the neural responses across participants. In contrast, the DYS group does not show the same regularity in neural responses, suggesting the corresponding mechanisms for recognizing, decoding, and reading letters are less fine-tuned in the DYS group. This latter finding has additive value to Breznitz’s Asynchrony Theory (Breznitz, 2008) which proposes that dyslexia is an outcome of the failure to synchronize the various brain entities activated during reading-related processes. Nevertheless, this asynchrony is also evident for children with dyslexia and their typically developing counterparts when the stimulus’ complexity increases – as occur in the rime or visual confusability. This finding underscores the need for additional neural resources to resolve the stimulus’s confounding elements (rime or visual). To that end, the Cumulative RAN-related neural congruency components, fail to capture a consistent neural-response across either of the groups.

### 4.2. Differences between DYS and CAC is the lasso-aggregate neural-congruency components

Although the simple aggregation of the top three neural-congruency scores revealed significant differences between groups under the non-confounding conditions, we hypothesized that an optimally weighted sum over all ten neural-congruency components would capture additional differences in neural activations between groups. Indeed, the sparse logistic regression classification revealed that an optimally weighted aggregation over the neural-congruency components differentiates between CAC and DYS across both confounding and non-confounding conditions. These results suggest that components beyond the three with the highest lambda values do capture neural activity relevant to the task. Moreover, the weights associated with each component differ in both amplitude and sign (i.e., they can contribute either positively or negatively to the sum). This finding suggests that the neural activations captured by each individual component might appear with different intensity and polarity in each group. For example, one component that might capture activity associated with character-disambiguation might exhibit stronger synchronicity in one group (i.e., contributing positively to the sum); however, another component might exhibit stronger synchronicity in the other group. Thus, simple aggregation of components might result in cancelling out this effect. Overall, our classification model suggests that individual components must be considered when analyzing neural activations.

### 4.3. Interpretation of the spatiotemporal profiles

Spatial (i.e., the forward model) and temporal profiles of the extracted components are depicted in Figure 4 for each condition. The forward model of the neural-congruency component #1 (i.e., the one with the highest lambda score) exhibits a similar topography across all four conditions; moreover, their corresponding temporal profiles show the neural activity is most strongly modulated at a time window of around 100 ms. The similarity suggests that component #1 captures neural activity common to all four conditions, albeit at different intensity levels. At the very least, this finding confirms previous evidence showing that processing complex features of textual stimuli is reflected in the electrophysiological responses around 100 ms after stimulus presentation (Hauk et al., 2006).

The temporal profile of components #10 shows similarity in waveform across the four conditions and a peak amplitude at around 100 ms following the fixations onset. Similarities in the spatial profiles are observed among some of the remaining components as well, although the indexing/ordering of those components varies among conditions. Such similarities suggest that the matching neural-congruency components likely capture neural activity originating from the same underlying source. The variation in the indexing is expected since the ranking of the components is established independently for each condition and depends on the relative strength of all the neural-congruency components in that condition. Interestingly, several projected temporal profiles in each condition display a stereotypical response consistent with neural activations often observed in traditional grant average Event-related Potential analysis (i.e., N/P100 and N170). For example, the temporal profile of component #9 shows an N170 waveform response with visible differences in amplitude at around 170 ms between CAC and DYS. Moreover, the forward model topography of this component shows it to emerge more strongly in electrodes over the left posterior-occipital regions. In the literature, the difference in N170 over the left-posterior occipital region is regarded as an electrophysiological marker of visual expertise (Varga et al., 2020). Particularly, children with a lower letter knowledge, as pre-readers, have shown reduced N170 amplitudes and delayed N170 latency compared to typical readers during letter-string presentations (Maurer et al., 2005). Therefore, component #9, extracted by our method, can be interpreted as potentially indicating a visual precursor to literacy resulting from familiarity with letter strings. Furthermore, negativity components at 170 ms have been associated with attention modulation (i.e., Kropotov, 2016), and in turn, attention represents a known latent common cognitive factor of RAN and reading (Papadopoulos et al., 2016). Therefore, part of the neural activity captured by the component could also reflect those distal processes. Moreover, several forward model scalp plots display topographies that often arise as the “forward problem” solutions to single-source dipole models, indicating that those components likely capture source-localized neural activity of different neural processes. Finally, components with high lasso coefficients (i.e., either positive or negative) capture underlying neural activity that more strongly differentiate between DYS and CAC. Thus, group differences among groups appear in the underlying sources modeled by those components.

Further studies could investigate these components in more detail and draw additional conclusions about the neural and cognitive processes that contribute to these differentiations. For example, an interesting expansion of these findings relates to examining the validity of the suggested neural-congruency components analysis against other more conventional EEG analyses. Given that the literature has only recently started to investigate the important information that the FRPs can provide to studying the RAN-reading relationship or other similar correlates of reading performance (e.g., Christoforou et al., 2021b; Fella et al., 2022), the present findings are considered a promising beginning of this quest. Another interesting expansion would be to examine the relationship of the extracted neural congruency components to eye-tracking-based metrics during RAN, such as the recently proposed entropy-based gaze time-series analysis on RAN (Wang et al., 2022).

In conclusion, the RAN-related neural congruency component, identified by our proposed method, carry information on the neural basis of naming speed that differentiates between children with dyslexia and their typically developing counterparts. The topographies of the resulting components suggest that each component likely captures source-localized neural activity corresponding to distinct neural processes. Moreover, neural differences appear to be distributed across several RAN-related Neural-congruency components but at different intensity levels. Therefore, optimally combining the RAN-related components using machine learning enhanced the power of analysis in identifying differences in both the confusable and non-confusable conditions, which have been missed by simple aggregation of the RAN-related Components. Our findings also support the Neural-congruency hypothesis (Christoforou et al., 2021b; Christoforou and Theodorou, 2021), indicating that neural activity elicited during cognitive tasks is more congruent among participants that have mastered the cognitive skills but less congruent otherwise. Finally, our proposed approach opens up new research directions in studying the neural underpinnings of naming speed and their association with reading performance and reading difficulties. For instance, until recently, evidence concluded that individuals with reading difficulties show increased reliance on inferior frontal regions of the reading network and right-hemisphere posterior regions (e.g., Richlan et al., 2011; Norton et al., 2015). The present findings show that several brain areas contribute to the execution of naming tasks which show similarities with reading tasks. Although simple at the surface level, RAN tasks are multi-componential, as is reading (Papadopoulos et al., 2016). Thus, we believe that the present method succeeded in better defining the properties of other processes (including those of reading) that RAN carries and how these are critical in determining naming speed’s influential role on reading performance. At the very least, capturing the RAN performance in the form of neural components helps us better understand the process involved in performing RAN tasks and explore some reasons for poor performance. Next, provided that the generated components will be further systematically tested against behavioral performance measures, the likelihood of deciphering issues relevant to the significant similarity of the RAN and reading measures, such as seriality, is possible (see Altani et al., 2020). Thus, in future work, we plan to further explore the spatiotemporal characteristics and brain sources of the RAN-related Neural-congruency components and the relationship between the neural underpinnings of naming speed - as captured by the Neural-congruency components- and reading difficulties, as well ex explore eye-tr.

## Data availability statement

The datasets presented in this article are not readily available because further analysis of the data is currently in progress. Requests to access the datasets should be directed to CC, christoc@stjohns.edu.

## Ethics statement

The studies involving human participants were reviewed and approved by the Cyprus National Bioethics Committee. Written informed consent to participate in this study was provided by the participants’ legal guardian/next of kin.

## Author contributions

CC conceived the original methodology, designed and developed the computational framework, carried out the implementation of the methods, data pre-processing, and analysis, and took the lead in writing the manuscript. MT contributed to the method’s implementation and designed the figures. CC, AF, and TP contributed to the RAN experiment and study design and data collection. TP contributed to drafting and editing the manuscript and to the interpretation of the results. All authors discussed the results and commented on the manuscript.

## Footnotes

1 Kronecker delta is defined as: δ_*ij*_ = 1 if *i* = *j*; delta δ_*ij*_ = 0 if delta *i* ≠ *j*
