# Supplementary material for: RAN-related neural-congruency: a machine learning approach toward the study of the neural underpinnings of naming speed
Source: Front Psychol. 2023 Jun 20;14:1076501. doi: 10.3389/fpsyg.2023.1076501 (PMC10319123; doi:10.3389/fpsyg.2023.1076501)
Supplement: Supplementary file 1 [file Data_Sheet_1.pdf]

Supplementary Material

1 Supplementary Tables and Figures

Table 1

Naming Speed Analysis of Behavioral data

	Groups					
	DYS		CAC		*F*	
	M	(SD)	M	(SD)		
Rime confusable	40.08	(10.89)	34.14	(6.79)	6.44	**
Rime non-confusable	43.45	(12.40)	35.60	(6.49)	9.36	**
Visual confusable	56.85	(13.53)	44.41	(9.97)	16.43	***
Visual non-confusable	49.35	(11.60)	39.13	(7.70)	16.17	***

Note. DYS: Group with dyslexia; CAC: Chronological age controls; M: mean Naming speed (reported in seconds); SD: standard deviation; ** p <.01; *** p <.001.

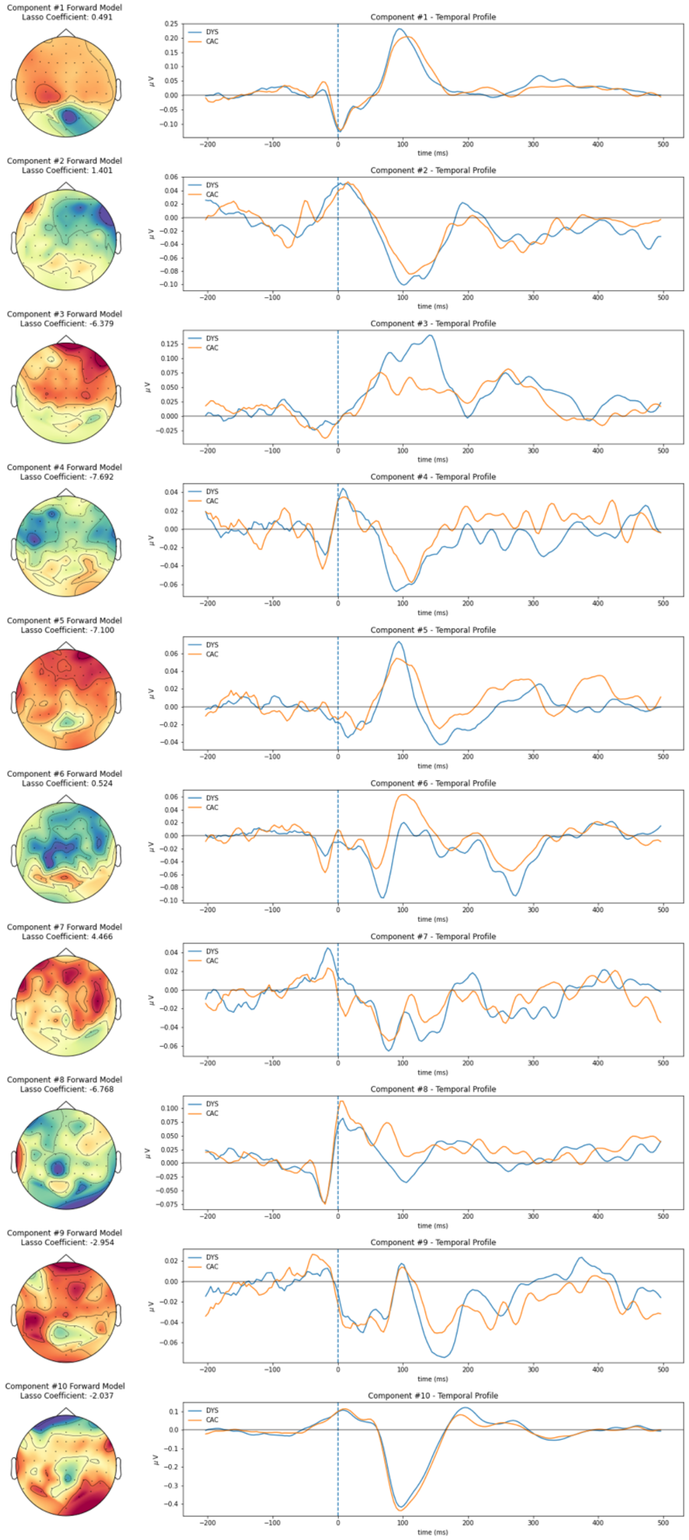

Supplementary Figure 1. Spatiotemporal profiles of RAN-related Neural-congruency components for the *rime-confusable condition*

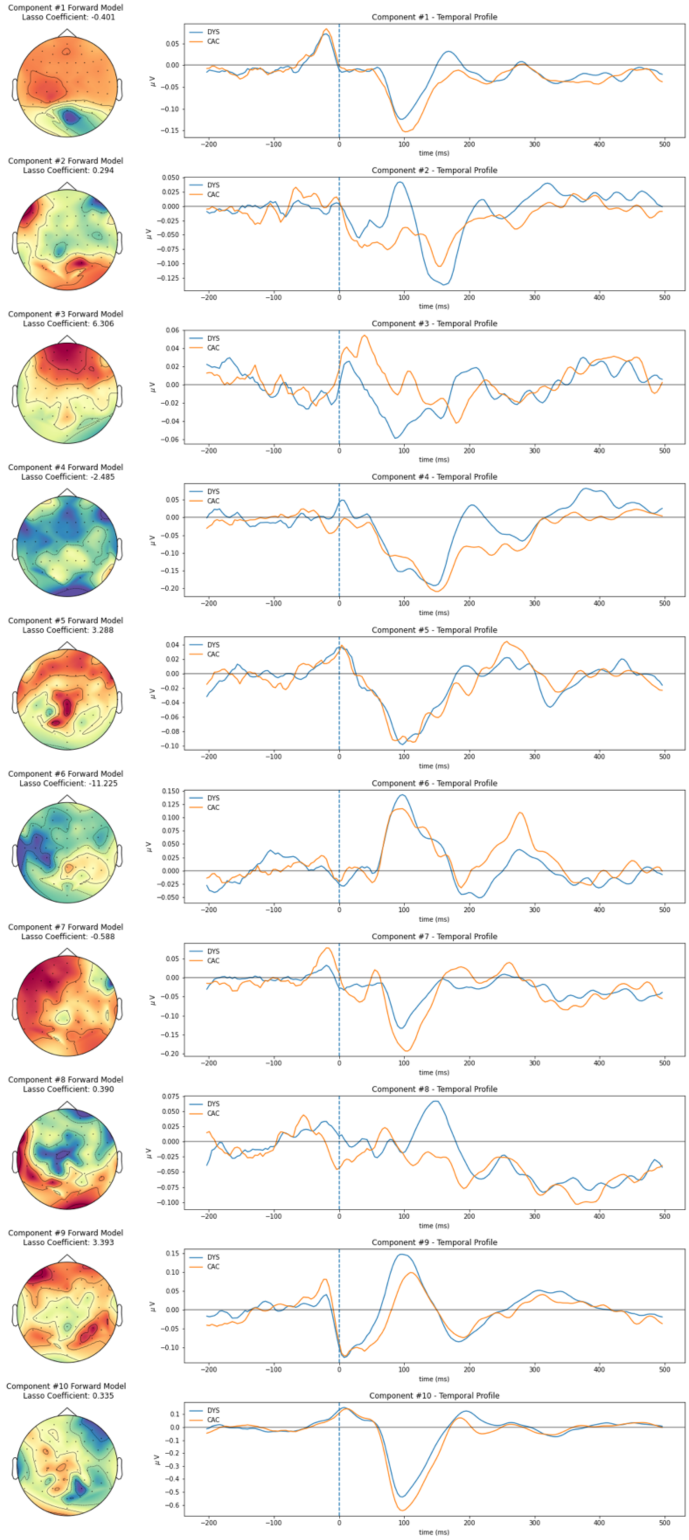

Supplementary Figure 2. Spatiotemporal profiles of RAN-related Neural-congruency components for the *rime-non-confusable condition*

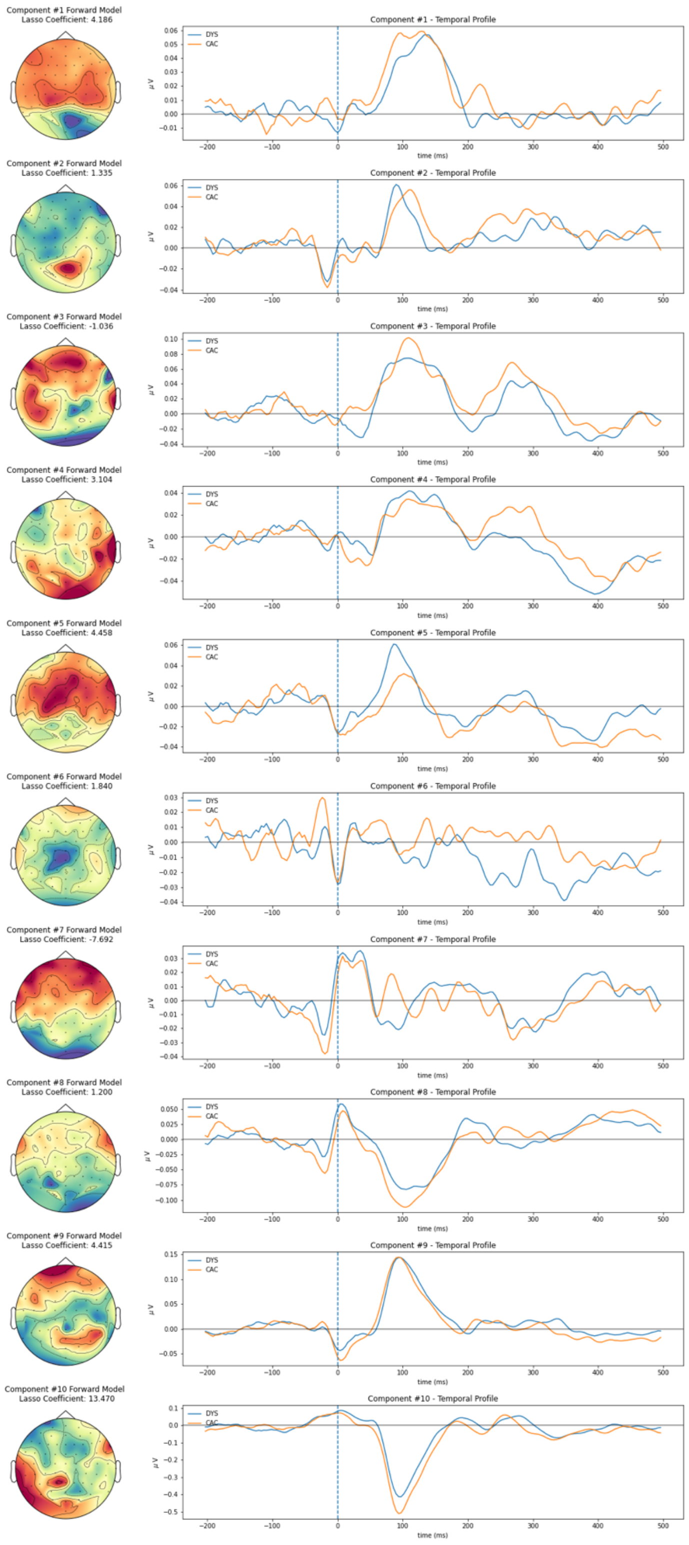

Supplementary Figure 3. Spatiotemporal profiles of RAN-related Neural-congruency components for the visual-confusable condition

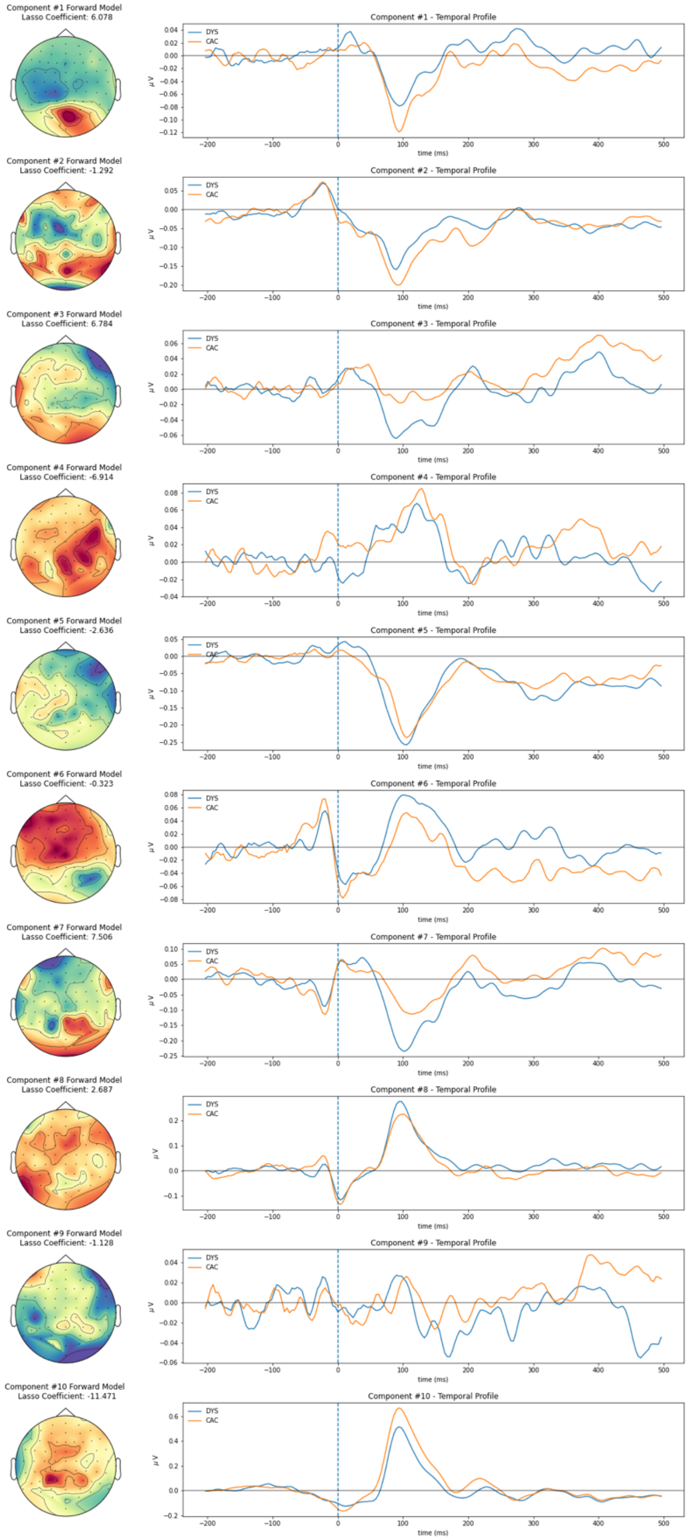

Supplementary Figure 4. Spatiotemporal profiles of RAN-related Neural-congruency components for the *visual-non-confusable* condition
